# Design and Implementation of an Energy-Efficient Weather Station for Wind Data Collection †

**DOI:** 10.3390/s21113831

**Published:** 2021-06-01

**Authors:** Padma Balaji Leelavinodhan, Massimo Vecchio, Fabio Antonelli, Andrea Maestrini, Davide Brunelli

**Affiliations:** 1 OpenIoT Research Unit, Fondazione Bruno Kessler, 38123 Trento, Italy; pleelavinodhan@fbk.eu (P.B.L.); fantonelli@fbk.eu (F.A.); amaestrini@fbk.eu (A.M.); 2 Department of Industrial Engineering, University of Trento, 38122 Trento, Italy; davide.brunelli@unitn.it

**Keywords:** weather station, wind data, energy neutrality, precision agriculture, meteorology

## Abstract

Agriculture faces critical challenges caused by changing climatic factors and weather patterns with random distribution. This has increased the need for accurate local weather predictions and weather data collection to support precision agriculture. The demand for uninterrupted weather stations is overwhelming, and the Internet of Things (IoT) has the potential to address this demand. One major challenge of energy constraint in remotely deployed IoT devices can be resolved using weather stations that are energy neutral. This paper focuses on optimizing the energy consumption of a weather station by optimizing the data collected and sent from the sensor deployed in remote locations. An asynchronous optimization algorithm for wind data collection has been successfully developed, using the development lifecyle specifically designed for weather stations and focused on achieving energy neutrality. The developed IoT weather station was deployed in the field, and it has the potential to reduce the power consumption of the weather station by more than 60%.

## 1. Introduction

Climate change can potentially disrupt whole national economies, with dramatic consequences for the lives and livelihoods of every human in this world. Weather patterns are changing, sea levels are rising, while exceptional weather events are becoming more and more extreme. Climate action represents one of the 17 Sustainable Development Goals (SDGs) of the United Nations’ 2020 report [1], while the farming industry globally feels the impact of climate change, through changing rainfall patterns, unseasonal weather, rising temperatures, and extreme climate events [2]. NASA’s evidences for this rapid climate change are compelling: the planet’s average surface temperature has risen about 1.18 °C since the late 19th century [3]. These mean changes in temperatures and precipitations, as well as more frequent abnormal weather events, affect crop yields, creating immense financial pressure [4]. In the European Union (EU) alone, 22 million people are employed directly in the farming sector and up to 44 million people rely on the food sector [5]. To worsen the situation, the use of excessive fertilizers and pesticides is becoming a common agricultural practice that is causing multiple farmlands issues, such as land degradation, nutrient loss, and biodiversity loss. In general, more sustainable practices for agricultural production and food systems are required to combat this dangerous escalation.

Variability of the weather conditions, even within small regions, has increased the demand for local sensor systems to monitor the crops with the objective of taking the correct decision at the right time. Today, sensing the local weather conditions plays a vital role in effective decision-making systems based on precision agriculture technology [6]. Precision agriculture is a management strategy to improve the production and quality of the crops through Information Technology (IT) [7]. It is focused on collecting data from the agricultural fields and using this data for analysis to improve the quality and the quantity of crop yields. The collected data can be then used to mitigate the crops from various adverse events, like undesirable climate, over usage of pesticides, diseases, and weeds, and take measures to reduce the impact of such events. In precision agriculture, wind data represents important information for estimating parameters, such as precipitation [8], soil health [9,10,11], and early-warning frosts [12]; therefore, such knowledge is a valid instrument for the farmer to reduce losses due to climatic factors. Furthermore, it is worth recalling that wind information is also useful in other sectors, such as energy generation [13,14,15], operation safety of Unmanned Aerial Vehicles (UAVs) [16], and even buildings [17]. Thus, the development of cost-effective and robust weather stations able to collect wind data in private fields is the need of the hour.

In response to this urgency of the agricultural sector, the Internet of Things (IoT) can be a trusted ally, as it has the potential to enable uninterrupted data collection from remote environments at relatively low costs [18]. Generally speaking, the demands of modern IoT-based deployments [19] are related to security, privacy, reliability, scalability, etc. However, when thinking about IoT devices as monitoring and actuation elements in agricultural and rural scenarios, even more elementary needs act as inhibitors of the IoT global adoption. Only to mention the most urgent ones: we require long-range, affordable, and power-aware radio communication technologies, the need for cheap yet robust hardware devices able to work outdoor and unattended for at least one yield season, the ease of installation and operations [20].

Notwithstanding these premises, in the last few years, the demand for modular, robust, and uninterrupted IoT-based weather stations is exploding. In the literature, there are different system architectures for weather data collection, as listed in Reference [21]. The wide spread of open-source IoT software platforms and technologies, as well as the availability of open-source hardware projects have highly reduced the development costs and the deployment efforts of personal IoT systems. Therefore, some of today’s IoT-based weather stations are cheap and easy to install and maintain [22], while they can collect data from the fields and transmit them to distant servers in an affordable way [23]. However, the majority of commercially available IoT-based weather stations fail to address the energy constraints, resulting in the limitation of the technology. The energy problem in wireless sensor networks, which includes IoT, is one of the significant barriers preventing the full exploitation of this technology [24]. Usually, researchers model the energy problem as an optimization problem, tackled in different ways that we can group into two major categories, namely:

- Hardware optimization:the correct selection and integration of hardware components (microcontrollers, sensors, and communication technology) plays a vital role in achieving hardware optimization. For instance, in the context of low-power wide-area network communication technologies, LoRa (acronym of Long Range) is often considered as a suitable choice to develop a remote weather station ([25,26]), although there are also other technologies observed in the literature, such as WiFi [27] and Xbee [28].
- Software optimization: the most used optimization strategy in this context is fine-tuning the operating system’s scheduling policy so as to reduce power consumption [29]. Other approaches include the adoption of smart software modules that, by avoiding the transmission of redundant values, can reduce the average data transmission rate, reducing the overall power consumption as a consequence [7,30,31].

The aforementioned works are focused only on the implementation of the weather station and fail to estimate the power requirements of the weather station. They try to reduce the power consumption by using low-power electronics or optimization algorithms but fail to consider the battery requirements. Some commercially available solutions for weather data collection rely on non-rechargeable batteries, so optimizing battery life is vital in these cases. Batteries pose environmental issues, as their proper disposal costs both time and money. Furthermore, weather stations are installed frequently in locations that are difficult to access. So, the labor costs of battery replacement are considerable. A battery-powered IoT weather station is introduced in Reference [32] bringing in the perspective of power consumption measurements in the weather station. However, most of the works dealing with the development of energy-neutral weather stations for precision agriculture is mere implementation and integration effort [21,27,28,33,34]. A holistic approach to solve the energy demand by using rechargeable batteries, low power electronics, optimization algorithms in embedded software as a whole package is not considered in the literature. Even if we consider weather stations endowed with rechargeable batteries and solar panels, we argue that saving energy by employing optimized embedded software is still a desirable feature. With more power efficiency, such weather stations will require smaller batteries and solar panels, which reduces the cost and improves the sustainability of the monitoring system. In this niching literature, we can find research efforts focused on achieving uninterrupted weather data collection using state-of-the-art energy harvesting and storage technologies.

We argue that a system-level view is required to design IoT devices that are energy-neutral [35]. This problem statement is complex and connected to different domains, such as energy harvesting, low power electronics, software engineering, meteorology, etc. So, systematic development is required, and this problem should be considered as a design problem rather than an optimization problem. Along this line, we recently introduced a systematic approach to the design of such an energy-neutral IoT weather station [36]. Following the weather station development lifecycle proposed in the previous work, in this paper, we develop, deploy, and profile from an energy consumption perspective a real-time wind weather station. Briefly, by extending our previous work, this paper proposes a new possibility to optimize the weather station’s energy requirements, thereby opening the perspective to energy-neutral weather stations. The main contribution of this paper is a complete design, development, and validation of an asynchronous optimization algorithm for a weather station with wind data collection. In addition, the energy profile of the weather station is studied, and its energy requirements are estimated.

The rest of this paper is structured as follows: Section 2 provides the rationale of this paper, with detailed information on setting the experiments required for the analysis, methods to validate the data from the experiments performed, and to interpret its results. Section 3 introduces the technical specifications of the anemometer for wind data collection, both from the software and hardware development perspectives. Then, Section 4 proposes a novel algorithm to optimize the energy consumption during wind data collection procedure, based on wind speed and direction. On completion of weather station development, it is necessary to estimate its energy requirements: In Section 5, the energy profile of the weather station is measured, and the energy requirements for uninterrupted operation of the weather station is estimated. In Section 6, the proposed framework is thoroughly validated in a simulation environment using a public available weather dataset from the National Renewable Energy Laboratory (NREL) and in real-time deployment. Finally, in Section 7, we draw our conclusions, briefly identifying the most promising future research paths.

## 2. Materials and Methods

This section summarizes our previous work also introducing the rationale of this paper. Its main goal is to provide an overview of the proposed approach and the planned experiments, as well as means to interpret the obtained results.

**Figure 1:**
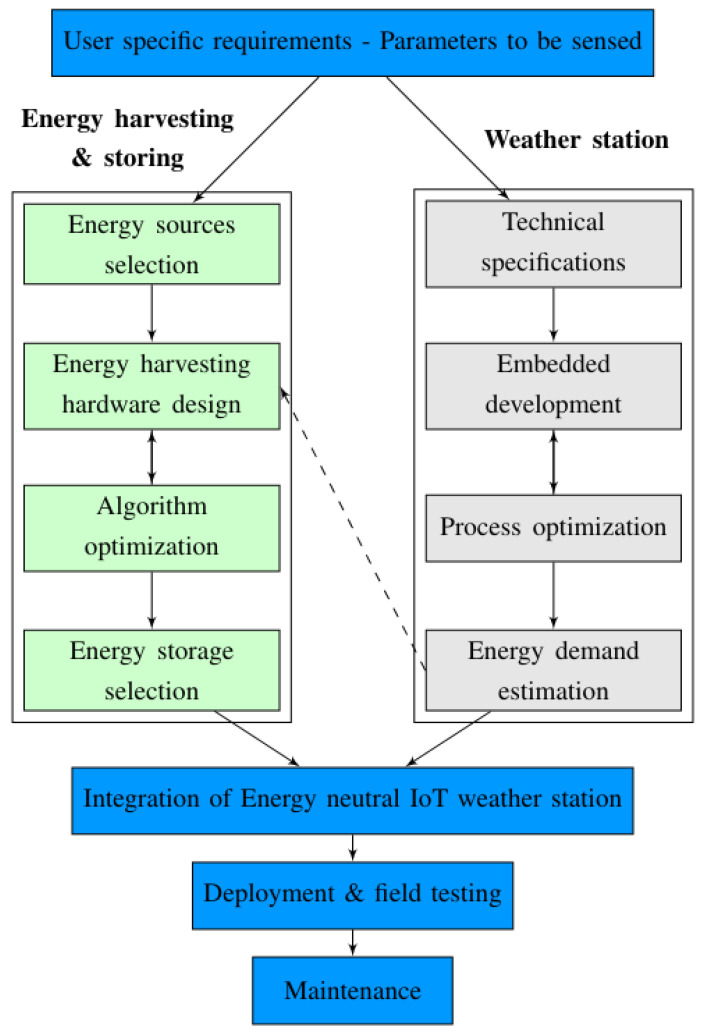
Development life cycle of IoT based weather station proposed in the previous work [36].

In our previous work [36], the intricate details of developing an energy-neutral weather station were studied. Specifically, we proposed a development life cycle that can be used to drive the design and the development of a real weather station, as shown in Figure 1.

The primary challenge of designing an energy-neutral weather station is to optimize all of its components and sensors in terms of energy consumption to reach neutrality. The resulting device can absorb or harvest enough energy from the environment (e.g., light, heat, radio waves, etc.) and generate the required electricity for its operation [36]. The first phase of the proposed life cycle consists of collecting the technical specifications, based on the user-specific requirements. For instance, if the user is a farmer who needs to measure a set of parameters, such as rainfall level or wind speed, then suitable sensors, like a rain gauge or an anemometer, have to be part of the weather station technical specifications. After finalizing the technical specifications, it is necessary to understand the sensor working and then interface it with the microcontroller through the embedded development and integration tools. This activity opens up new opportunities to optimize the energy consumption of the employed sensors, which is the objective of the next step, namely the process optimization phase. The last phase consists of estimating the energy demands based on the developed weather station to determine the design parameter of the energy harvesting hardware with optimal accuracy.

Strictly following this methodology described in this paper, we focus on developing a weather station endowed with an anemometer sensor for collecting wind data. Cost-effective and open-source hardware and software resources are used for development, narrowing down the search for available options. Wind speed measurements of the anemometer sensor depend on the sample period selected; therefore, a detailed analysis on the selection of sample period is studied. Later, a simple interface is implemented to collect the data from the anemometer. This data is beneficial to understand how the anemometer sensor works. In addition, the relation between the parameters of wind speed and wind direction is studied. The energy perspective of the weather station is evaluated, and, finally, a new algorithm to collect the wind speed and direction is proposed, implemented, and tested. The primary objective is to find the valuable data points and send those data points through asynchronous wireless transmissions instead of sending all the data at a fixed sample rate (i.e., in a synchronous way). Briefly, it is the end-user that, by setting a wind speed threshold depending on the wind conditions of the deployment site and/or the reconstruction error of the wind signal that his application can tolerate, trades-off power consumption of the weather station and the accuracy of the monitored phenomenon. The developed system only transmits significant data points, dropping the ones below the set threshold. Values below the threshold limit are not stored in the embedded system.

First of all, to validate the proposed asynchronous algorithm, real-time deployment of the developed weather station is planned. However, to accurately validate the proposed asynchronous approach, we need years of wind data. Therefore, we resort also to a publicly available dataset, namely the National Renewable Energy Laboratory (NREL) dataset. This wind dataset containing information about wind speed and direction is used as input data for the proposed algorithm to have a more extensive simulation campaign. The asynchronous algorithm focuses on reducing the number of transmissions which directly reduces the amount of energy consumed by the weather station. So, this analysis does not provide the exact details of how much energy can be saved by the proposed algorithm, but it provides proof of concept by counting the reduced number of transmissions.

## 3. Development of Weather Station for Wind Data Collection

In this section, the development life cycle for the proposed weather station is introduced. Briefly, as shown in Figure 1, the development life cycle consists of 4 steps: technical specifications, embedded development, process optimization, and energy demand estimation. The first two steps of this life cycle will be elaborated in this section, while the remaining two will be detailed in Section 4. In each step, the energy perspectives are investigated to direct this activity towards developing an energy-autonomous weather station.

The first step is to capture the user requirements and convert these user requirements into technical specifications. The second step is focused on developing the embedded system that comprises both the hardware and software design followed by the development. So, the hardware design of the weather station is a conversion of the user requirements. After completing the hardware development, the embedded software development is performed, focusing only on interfacing the peripherals.

### 3.1. Hardware Design

The primary user requirement for a weather station is the ability to collect wind data (in terms of both wind speed and direction) from remote agricultural fields, with low impact in terms of cost, installation and maintenance. Another stringent requirement is the application longevity: the weather station should be operational even when deployed in a harsh outdoor environment without any human intervention for at least one crop season. Therefore, low-power electronics devices are selected to achieve optimal energy usage. A cheap and low-power embedded system consisting of the following elements is derived to address the aforementioned critical requirements.

1. a cup anemometer (Davis DS6410);
2. a low power MCU (Arduino Pro mini);
3. a LoRa Communication Technology module (Mini-Lora v1.1a).

The requirements of a wind sensor are robustness and the ability to measure wind speed and wind direction. DS6410 anemometer, manufactured by Davis Instruments, fits the requirements; hence, it was selected as the wind sensor [37]. This anemometer transduces the wind direction by means of a wind vane connected to a linearly variable electrical resistance within the range of 0–20 KΩ. The resistance value provided as an analog output will change according to the actual wind direction. For such A/D conversion, the 10-bit ADC internal to the MCU will be used. More in detail, the wind vane is calibrated from the factory to output a value of 10 KΩ to represent the South direction with an angle of 180° meaning that East, North, and West correspond to values-angles pairs of 15 KΩ-90°, 0 Ω-0° (thanks to a dead band around the North direction) and 5 KΩ-270°, respectively. Conversely, the wind speed is digitally transduced by the anemometer by means of three wind cups that are free to rotate in the presence of wind. More in detail, each full revolution of the wind cups close a mercury switch, hence producing a digital pulse as output. The number of pulses can be converted into actual wind speed using the conversion formula provided in the device datasheet:

(1) $$v=\frac{p\times 2.25}{T},$$

where *v* is the wind speed measured in miles per hour (mph), *T* is the sample period in seconds (s), and *p* is the number of full pulses measured within the sample period *T*. It is important to note the value of a number of pulses measured within the sample period (*p*) will be a non-negative integer.

**Figure 2:**
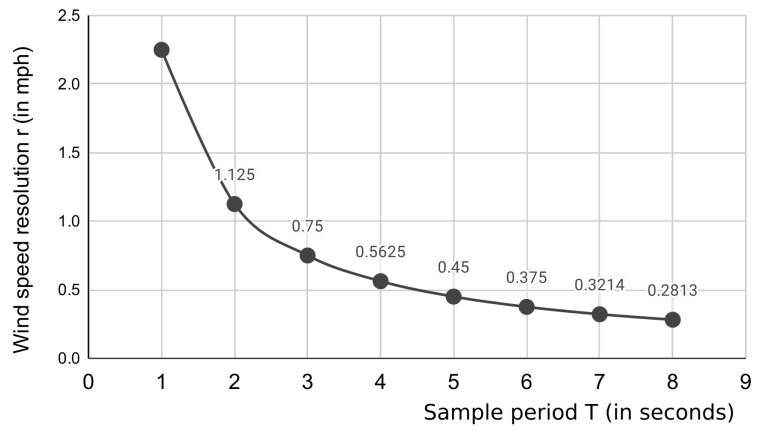
Sample period (T) vs wind speed resolution (r).

From Equation (1), we observe that *T* proportionally affects the wind speed resolution. For instance, if the sample period selected for an application was 1 s, recalling that *p* can assume only non-negative integer values, the minimum speed measured would be 2.25 mph, and multiples of such a value could be measured and reported by the sensor. We say that the sensor resolution *r* is equal to 2.25 mph. The opposite is also true: the more we increase *T*, the *r* becomes small and more precise, as depicted in Figure 2. Clearly, *T* directly affects the power consumption of the weather station, as the latter has to stay active during the whole measurement period to count the number of pulses from the sensor. Therefore, a suitable trade-off between power consumption and sensor resolution has to be reached. For our experiments, we set T=5 s to measure wind speeds that are multiples of r=0.45 miles per hour, which is a good trade-off for the types of winds characterizing our deployment field (see Section 6).

Regarding the communication unit, we opted for the Mini Lora Node (https://github.com/hallard/Mini-LoRa, accessed on 30 May 2021). Briefly, it is an open-source hardware project providing the interface between the RFM95 Low Power Long Range Transceiver Module (https://www.rfsolutions.co.uk/downloads/1463993415RFM95_96_97_98W.pdf, accessed on 30 May 2021) and the Arduino Pro Mini through a Serial Peripheral Interface (SPI) on a single PCB, with a dedicated room for various types of battery holders, such as AA, A, 18650. With this integration board, the selected MCU could be easily connected to the radio module, without affecting the other pins of the board, hence making the prototyping and testing phases straightforward. Specifically, only pins A0, D1, D7, D8, D10(SS), D11(MOSI), D12(MISO), D13(SCK) of the Arduino Pro Mini were used to interface it with the RFM95 module of the integration board.

Regarding the IoT infrastructure, a LoRaWAN gateway was installed in our premises to collect the data sent from the weather station, while the data collected was stored using a Raptorbox instance (https://smks.fbk.eu/it/results/raptorbox-the-iot-platform, accessed on 30 May 2021), which is an open-source IoT platform we have developed for rapid prototyping of IoT applications purposes.

**Figure 3:**
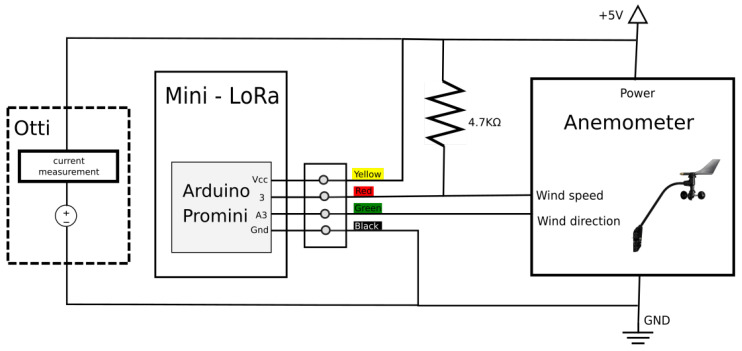
Anemometer connection circuit.

The complete circuit diagram for connecting the anemometer to the microcontroller board is depicted in Figure 3. More in detail, the anemometer was interfaced with an Arduino Pro Mini [38] development board and then deployed outdoor. The latter is based on the ATmega328P [39] microcontroller with a working frequency of 16 MHz, manufactured by Microchip [40] and endowed with 32K bytes of in-system self-programmable flash program memory and 1K bytes of EEPROM.

We selected this board for our application because of its reduced cost, small size, and its appealing low power capabilities. ATmega328P is capable of operating at a low current of 1 A in the power-down mode and 1.5 mA in the active mode (3 V@4 MHz configuration). Moreover, as a member of the Arduino open-source ecosystem, it was the perfect match for our fast-prototyping needs. The ATmega328P can operate from 2.7 V to 5.5 V and its power consumption varies based on the operating voltage, as well as the clock frequency. For our application, a 3.3 V power supply and 8 MHz clock frequency were selected.

Regarding the wiring, the anemometer came with an RJ11 male connector; therefore, a suitable female port was used to connect the sensor to the board. Specifically, the four lines of the connector (yellow, black, green, and red) were connected to the VCC, GND, analog input 3 (A3, for wind direction), and digital input 3 (D3, wind pulses) of the board, respectively. Then, an Otii Arc device from Qoitech [41] was used as a constant, linear power supply to power the circuit. The latter is a smart power meter, so, as done in Reference [23,42], we used it also to measure, profile and log the absorbed currents during the experiments at very high resolution and sample rate.

### 3.2. Embedded Software Design

To develop an embedded system that is energy efficient, the hardware and the software play a key role [35]. Without optimizing the software design, it is difficult to handle the embedded system’s constraints and achieve optimal usage of resources. It is required to explore the weather station’s functionalities before developing the algorithm and embedded code. The three major functionalities of the weather station are as follows:

1. Collect the wind speed data using the number of pulses.
2. Collect the wind direction data using the analog to digital converter.
3. Schedule the collected wind speed and direction data for transmission to the LoRa server.

**Figure 4:**
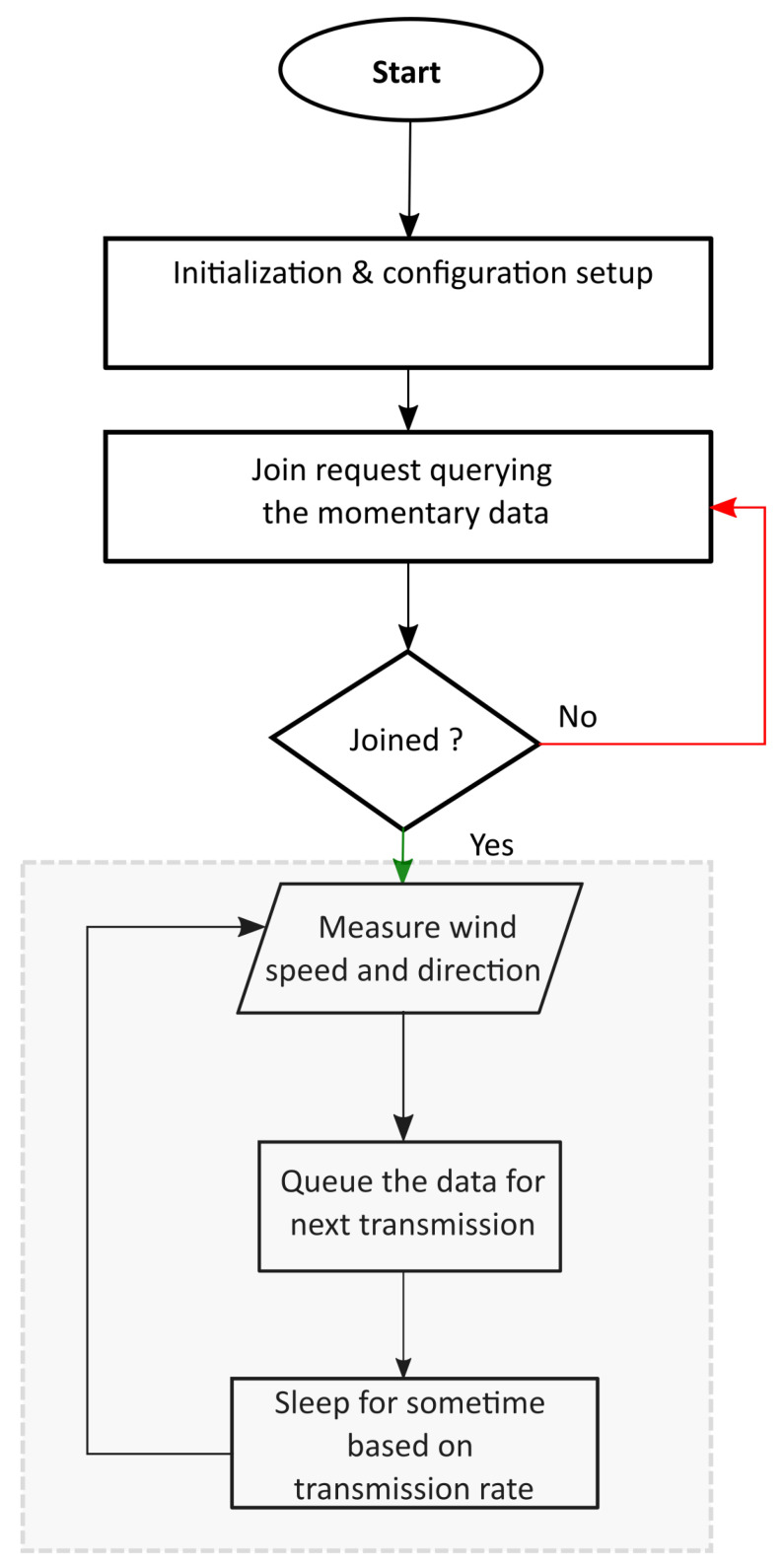
Basic algorithm for wind data collection.

Based on these three functionalities, an algorithm was developed to acquire the data from the anemometer and send it to the LoRa server based on the transmission rate requirements. The algorithm flow chart is available in Figure 4. Initially, the weather station is powered up and connected to the server. Till the joining with the Lora network is successful, the weather station retries. On successful joining, the rest of the activities are planned. The internal ADC is used to convert the voltage across the linearly variable resistance into a digital value, as mentioned in Section 3. This digital value represents the wind direction, and it is mapped to degrees by the MCU. A software timer library and an external interrupt of the MCU are employed to measure the wind speed. The pin3 of the MCU counts the number of pulses received by the anemometer using the external interrupt. As mentioned in Section 3, the timer module of the MCU uses the 5 s sampling time configuration and, once this period is over, the number of pulses measured is used to calculate the wind speed. Wind data consisting of the wind direction and speed are queued for the next transmission. The weather station is switched to sleep mode for a time calculated from the transmission rate requirements. Since the wind data is momentary, it is essential to have low sleep time to capture sudden gusts.

Open-source libraries and tools were used to develop the embedded code. Arduino Pro Mini was programmed using PlatformIO (https://platformio.org/, accessed on 30 May 2021) which is a cross-platform, cross-architecture, multiple framework, professional tool for embedded development. Arduino-LMIC (https://github.com/matthijskooijman/arduino-lmic, accessed on 30 May 2021) library was used to program the LoRa interface with the Arduino Pro Mini. The Arduino-LMIC library is a simple event-based programming model in which all the functions are modeled as events or jobs. It has a built-in run-time environment to take care of timer queues and job management. Finally, the open-source library TimerOne (https://www.arduino.cc/reference/en/libraries/timerone/, accessed on 30 May 2021) was used to develop the interface with the anemometer sensor and the Arduino Pro Mini.

## 4. Process Optimization

Most of the recent related activities are focused on reducing the transmission rate at the source, as transmitting data wirelessly is, generally, the most energy demanding task for the sensing device [30,43]. Alternatively, one can use suitable power-aware data compression schemes to reduce the average packet size, as done in Reference [31,44]. Reducing the transmission rate is a viable strategy, but it could be worth tackling the challenge with more understanding of the measured parameters, for instance, the one we describe in the following.

Briefly, measuring the wind speed with the anemometer described in Section 3 is an energy hungry task. In addition, the energy required to transmit this data is the most energy consuming task of the whole weather station. It is pointless to collect the wind direction data when there is no wind, and so the whole data transmission is not adding value to the weather station. Therefore, our sensing board is programmed to periodically execute a check for the presence of wind (that is if a pulse on its dedicated pin is detected). After this check, if wind is detected, the MCU enters a speed measurement phase during which it updates the counter through the external interrupt service routine. The measured wind speed and wind direction are transmitted only if the wind speed is greater than the threshold wind speed set by the user.

Our main idea is to reduce the power consumption of the sensing device by collecting and sending only valuable information rather than sending all the information. This problem has been addressed, and a novel asynchronous optimization algorithm to collect the data from the anemometer is proposed in this section. The algorithm works based on the value of two flags, namely the rotation flag and the data ready flag. The functionalities of these flags are listed below.

- Rotation flag is set —Whenever the anemometer sends the pulse to the MCU using the external interrupt service routine. This action wakes up the MCU from sleep.
- Rotation flag is reset—After the variable rotations is incremented. If the rotation happens for the first time, this activates the timer to keep track of the sampling period.
- Data ready flag is set—When the timer completes its sampling period, it sets the data ready flag in the timer interrupt service routine.
- Data ready flag is reset—After the wind data is scheduled to be transmitted, all the variables and flags are reset. During this time, the data ready flag is also reset.

**Figure 5:**
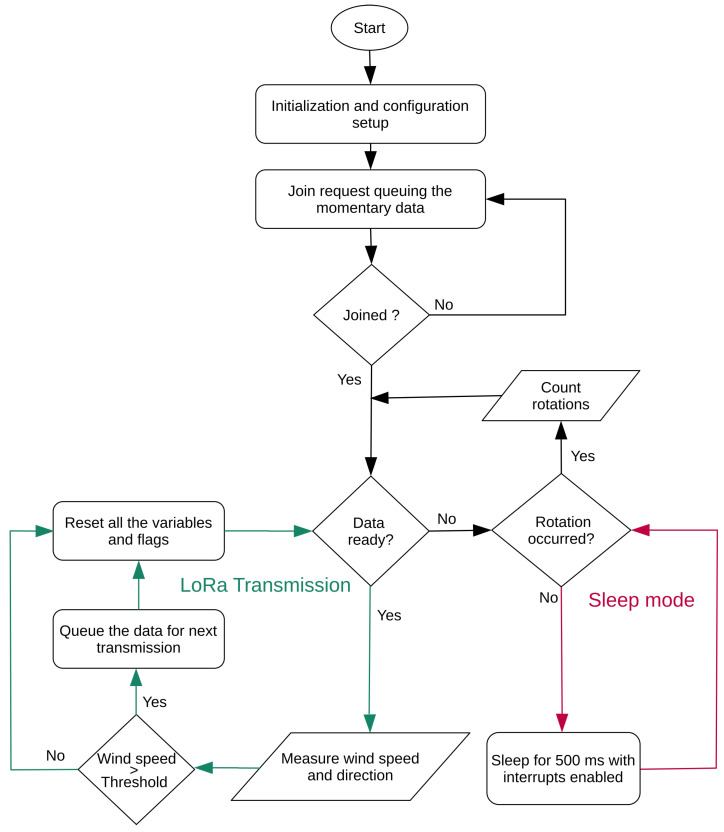
Asynchronous optimization algorithm for wind data collection.

From Figure 5, there are two main activities in the optimization algorithm that deserve attention. The first one is the LoRa transmission cycle, and the other one is the sleep mode (they are marked in green and red in Figure 5, respectively). Initially, there is no wind data available; therefore, the MCU is put into sleep mode. In the event of wind, the anemometer cups start rotating; this rotation is transformed into a pulse by the anemometer and sent to the MCU causing the external interrupt to happen. This interrupt sets the rotation flag and wakes up the MCU. Simultaneously, the number of pulses are counted and the count down starts for the sampling time *T*, 5 s, in our case. Only when the sampling time is over, the data ready flag is set, and the MCU starts to calculate the wind data from the available anemometer readings. The calculated values are scheduled to be sent if the wind speed is greater than the threshold wind speed and stored in the transmission queue. All the values and flags are reset, and the MCU is ready for collecting the next wind data. The pseudo-code of the described algorithm is depicted in Listing 1.

**Listing 1:** Pseudo code for Asynchronous optimization algorithm.

wind data collection function
if data ready flag is set
Stop the timer
Calculate the wind speed using the rotations value
Calculate the wind direction using the ADC
if the wind speed is greater than the threshold
Queue the wind data for the next transmission
Reset variables wind speed, wind direction and rotations value
Reset rotation flag and data ready flag
Reset timer
else
if the rotation flag is set
if rotation occurs the first time after reset timer
Start the timer
Schedule the wind data collection job
else
while rotation flag is reset
Sleep for 500~milliseconds

timer interrupt service routine
if sampling time is over
Set data ready~flag

external interrupt service routine
Increment rotations value
Set rotation flag

In our framework, the optimization of energy consumption is achieved by using the low power operation and the proposed asynchronous optimization algorithm. The main functionality of the algorithm is to compare the collected weather parameter and trigger transmission only when it is greater than the threshold. This kind of transmission is asynchronous and not based on any time period; hence, it can be adapted to other weather parameters which are random by nature. In the next section, the energy perspective of the weather station is explored.

## 5. Energy Demand

**Figure 6:**
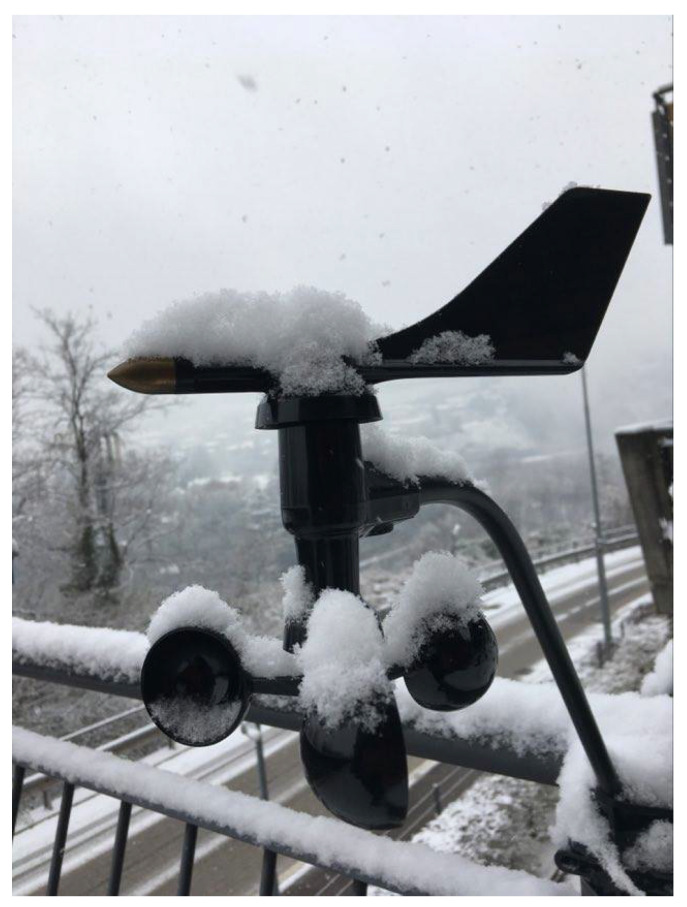
Anemometer deployed in the outdoor environment.

The weather station was deployed in an outdoor environment as depicted in Figure 6 and an Otii Arc device was used to profile and record the weather station’s power consumption during its operations. In this section, the energy consumption details of the wind weather station are presented and discussed. Briefly, by using the Otii device and the corresponding telemetry software, we could accurately measure the current, time, and energy values summarized in Table 1.

More in detail, a transmission cycle consists of an active phase, a transmission phase, and a reception phase, all measured using the Otii device. One transmission cycle took on average 6000 ms. First, the weather station switches to the active state from the sleep mode. Then, it transmits the data, waits for data reception, and, finally, upon data reception, switches back to the sleep mode. In the Otii software, a specific data transmission telemetry chuck was selected to get measures in terms of maximum current, average current, and energy consumption during that data transmission. The weather station operates in the active state most of the time during the transmission cycle (i.e., 5690 ms) absorbing, on average, 4.05 mA of current. There is a spike in the current values with a peak of 126 mA, with an average current absorption of 124 mA during the data transmission that has a short duration of 60 ms. The average current absorbed during the data reception phase is 15 mA, lasting the latter more than four times the duration of the transmission one (i.e., 250 ms). The device remains in sleep mode for the remaining time, absorbing negligible currents (i.e., 273 µA).

It is possible to estimate the energy requirements of the weather station for a period of time using the current consumption values measured using the Otii device. Since data transmission is the most significant power consumption factor, different scenarios are created to estimate the maximum and minimum power required to operate the weather station. As we have measured that one transmission is about 6 s, we set the active cycles as multiples of 6 s. Based on the different data rates, detailed power consumption is depicted in Table 2. The maximum energy consumed for one day is 4720.8 mJ, which was recorded when ten data is sent per minute. When the weather station is in complete sleep mode without transmitting any data for one day, the energy consumed by it is 54.06 mJ. It is important to notice that these are estimated values without considering the losses during long operation times. The data transmission function of the weather station plays an important role in understanding the weather station as it consumes more energy than other functionalities. From Table 2, it is clear that optimizing the data transmitted will, in turn, optimize the power consumption of the weather station. Therefore, the proposed asynchronous optimization algorithm has the potential to reduce the number of transmissions that the weather station has to execute to transfer useful data to a distant server for storage. However, such an algorithm has to be validated with weather data to understand the actual impact of the algorithm on the whole application lifetime. This will be the objective of the next section.

## 6. Algorithm Validation and Field Testing

In this section, the performance of the asynchronous optimization algorithm will be validated first against simulated data (using the weather dataset available from the National Renewable Energy Laboratory, NREL) and then assessed against data collected from the deployed weather station in our test site.

### 6.1. Validation of Asynchronous Optimization Algorithm

**Figure 7:**
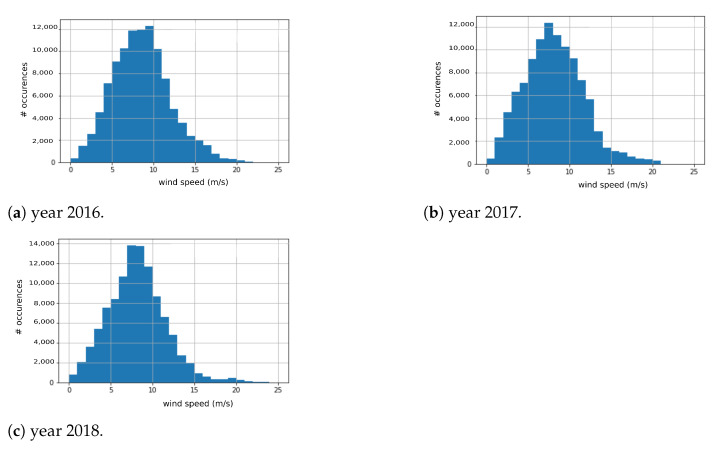
Distributions of the wind data.

For the validation, three years (from 2016 to 2018) of wind speed and direction data collected from the NREL Offshore NW Pacific Dataset is employed (https://developer.nrel.gov/docs/wind/wind-toolkit/offshore-nw-pacific-download/, accessed on 30 May 2021). More in detail, it is a 20-year wind resource dataset for offshore NW Pacific located in Canada at 48.76° latitude and −130.76° longitude, acquired at a height of 10 m from the ground, with a sampling period of 5 min (that is 288 samples per day, or 105,120 samples per year). We assume that this data is collected and processed by our asynchronous optimization algorithm setting two threshold values, namely $th_{1}=2$ m/s and $th_{2}=5$ m/s. The data distribution of each year wind data is available in the histograms shown in Figure 7.

The threshold wind speed data is a crucial parameter that can be decided based on the user requirements. Especially in the agriculture fields, the farmers can optimize it based on the nominal wind speed values for their region. As an example, out of the year 2016’s 105,120 data points, 16,086 data points were less than or equal to $th_{2}$ (that is, 15% of the total). For the sake of completeness, Table 3 reports for each year, the number of data points with a wind speed less than $th_{1}$ and $th_{2}$ ($R_{1}$ and $R_{2}$, respectively), together with their compression rates ($CR_{1}$ and $CR_{2}$, respectively), calculated as the fraction of the number of reduced transmissions $R_{i}$ with respect to the original (uncompressed) transmissions *N*, expressed in percentage [45] (see Equation (2)).

(2) $$CR=\frac{R}{N}\times 100.$$

This metric can be used to evaluate the impact of our asynchronous optimization algorithm, as it represents the percentage of transmissions reduced which, in turn, will reduce the energy consumption. On average, the asynchronous optimization algorithm reduces the transmissions by 5.7% and 17.83% when the threshold wind speed is set to 2 m/s and 5 m/s, respectively. This performance will change based on the geographical locations, as well as the selection of the threshold wind speed made by the user.

### 6.2. Field Testing Results

**Figure 8:**
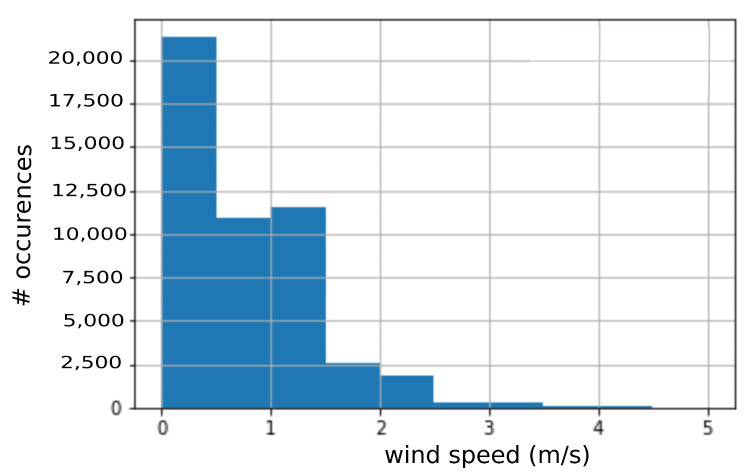
Nine days field testing results.

Experiments were conducted to evaluate the performance of the asynchronous optimization algorithm for wind data collection. The anemometer was deployed in an outdoor environment in Povo, Trento, Italy (46.07° latitude, 11.15° longitude), with the proposed algorithm and data was collected for nine consecutive days starting, from 22 November 2020, as shown in Figure 6. The anemometer weather station was configured to send data every 12 s, which is mid-level data rate, as mentioned in Table 2. All the data points were sent by the weather station to a Raptorbox instance for persistent storage. The data set was later downloaded from the server to analyze the performance of the anemometer weather station. The distribution of collected data is shown in Figure 8 as a histogram with a bin size of 0.5 m/s. From the figure, it is clear that the considered location has weak wind speed activity without any sudden gust; therefore, two threshold wind speeds were taken at 0.5 and 1 m/s to analyze the impact of the asynchronous optimization algorithm. It was computed that the compression rate was 43.42% for a threshold wind speed of 0.5 m/s and 65.72% for a threshold wind speed of 1 m/s. From this experiment, it is evident that the proposed algorithm is useful to reduce the number of transmissions which, in turn, reduces the power requirements of the weather station.

Considering the results from the NREL dataset and those of our real testbed, we conclude that the proposed asynchronous optimization algorithm is beneficial to the wind station as it guarantees a reduced number of transmissions with respect to conventional operations. Moreover, in the latter case, the energy consumption would be the same for each monitored day, as the wind station has to transmit at a fixed sample frequency. On the contrary, the proposed algorithm guarantees that only significant data points are transmitted, giving the user the full freedom to set the threshold above which data points start becoming significant, thus worth being sent. As an example, during our testing campaign in Trento, Italy, we could verify that several data points represented wind speeds less than 1 m/s (see the histogram of Figure 8) meaning that a threshold of 1 m/s is reasonable to meet a good tradeoff between accuracy of the monitored phenomenon and power consumption of the monitoring device. Clearly, moving our deployment to regions characterized by higher wind speeds would require changing the threshold accordingly, as we have shown with the NREL dataset. A promising research activity we are currently focusing on considers the adoption of meta-heuristic optimization tools (i.e., Multi Objective Evolutionary Algorithms, Taboo Search, Harmony Search, and Simulated Annealing, as we have already done in different application domains [46,47]) to more efficiently explore this complex solution space. However, this topic goes beyond the scope of this paper, so we leave it as future work.

## 7. Conclusions

In this work, a novel asynchronous optimization algorithm for weather stations was designed and developed while executing the development lifecycle of the weather station introduced in our previous work. Following the rapid prototyping principles and methodologies, a famous open-source embedded development platform, namely an Arduino Pro mini-board, was used to develop the proposed weather station. It was deployed in the field to collect real-time wind data. Later, analyzing the collected wind data lead to the design and development of a novel optimization technique. The weather station is optimized using the developed algorithm that maximizes the operation time of the weather station, minimizing the energy requirements by transmitting only the valuable data. Understanding the weather station’s energy requirements is the starting point to develop an energy-neutral weather station. It is possible to estimate the energy harvesting capacity from the weather station’s energy requirements, which will be instrumental in deciding the energy storage sizing.

In our future works, analysis of the asynchronous optimization algorithm in real-time, energy harvesting, and storing part of the development lifecycle, which is under development, will be explored. An experiment to collect the data from two weather stations, one using the asynchronous optimization algorithm, deployed at the same geographical location, is planned to be conducted. These data points will be statistically analyzed and compared with each other to prove that the data transmitted by our algorithm does not lose significant data points. Additionally, the data will be used as an input in weather prediction algorithms, and the variation in their prediction results will be analyzed to understand the impact of our algorithm. Another promising future direction deals with comparing energy performances of more power-aware embedded micro-controllers, such as the ones from the Texas Instruments MSP series, STM32 Arm Cortex MCUs, and Espressif ESP32 development kits. The objective, in this case, is to consider the cost of the hardware and the operations (e.g., type of batteries, size of solar panels, etc.) within the technical specifications phase.

## Figures and Tables

**Table 1:** Energy profile of weather station for one cycle of active and sleep modes.

State	Duration (ms)	Current (mA)	Power (mW)	Energy (mJ)
Active	5690	4.05	13.365	76.05
Transmitting data	60	124	409.2	24.55
Receiving data	250	15	49.5	12.375
Sleep mode consumes 273 µA of current and the power is 900.9 µW				

**Table 2:** Energy consumption profile of the test IoT device for one day.

Data per min	Duration (s)		Energy Consumption (mJ)		Total Energy (mJ)
	Active	Sleep	Active	Sleep	
0	0	60	0	54.06	54.06
1	6	54	472.1	48.65	520.75
2	12	48	994.2	43.25	1037.45
3	18	42	1416.2	37.84	1454.04
4	24	36	1888.3	32.44	1920.74
5	30	30	2360.4	27	2387.4
6	36	24	2832.5	21.6	2854.1
7	42	18	3304.6	16.22	3320.82
8	48	12	3776.6	10.8	3787.4
9	54	6	4248.7	5.4	4254.1
10	60	0	4720.8	0	4720.8

**Table 3:** Impact of asynchronous optimization algorithm on the 3-year NREL dataset.

Year	N	$R_{1}$	${\mathrm{CR}}_{1}$	$R_{2}$	${\mathrm{CR}}_{2}$
2016	105,120	4438	4.22%	16,086	15.3%
2017	105,120	7341	6.98%	20,781	19.77%
2018	105,120	6450	6.14%	19,371	18.43%

## Data Availability

Not applicable.
